# A Self-Powered, Highly Embedded and Sensitive Tribo-Label-Sensor for the Fast and Stable Label Printer

**DOI:** 10.1007/s40820-022-00999-y

**Published:** 2022-12-31

**Authors:** Xindan Hui, Zhongjie Li, Lirong Tang, Jianfeng Sun, Xingzhe Hou, Jie Chen, Yan Peng, Zhiyi Wu, Hengyu Guo

**Affiliations:** 1 School of Physics, Chongqing University, Chongqing, 400044 People’s Republic of China; 2 School of Mechatronic Engineering and Automation, Shanghai University, Shanghai, 200444 People’s Republic of China; 3 Beijing Institute of Nanoenergy and Nanosystems, Chinese Academy of Sciences, Beijing, 100101 People’s Republic of China; 4 College of Physics and Electronic Engineering, Chongqing Normal University, Chongqing, 401331 People’s Republic of China; 5 Electric Power Research Institute, State Grid Chongqing Electric Power Company, Chongqing, 401123 People’s Republic of China

**Keywords:** label-sensor, Infrared sensor, Triboelectric nanogenerator, Self-powered, Embedded sensor

## Abstract

Label-sensor is an essential component of the label printer which is becoming a most significant tool for the development of Internet of Things (IoT). However, some drawbacks of the traditional infrared label-sensor make the printer fail to realize the high-speed recognition of labels as well as stable printing. Herein, we propose a self-powered and highly sensitive tribo-label-sensor (TLS) for accurate label identification, positioning and counting by embedding triboelectric nanogenerator into the indispensable roller structure of a label printer. The sensing mechanism, device parameters and deep comparison with infrared sensor are systematically studied both in theory and experiment. As the results, TLS delivers 6 times higher signal magnitude than traditional one. Moreover, TLS is immune to label jitter and temperature variation during fast printing and can also be used for transparent label directly and shows long-term robustness. This work may provide an alternative toolkit with outstanding advantages to improve current label printer and further promote the development of IoT.

**Supplementary Information:**

The online version contains supplementary material available at 10.1007/s40820-022-00999-y.

## Highlights

- A self-powered and highly sensitive tribo-label-sensor is proposed as the substitution of infrared sensor for addressing current issues in label printer.
- Tribo-label-sensor shows higher integrability, sensitivity, reliability and universality than traditional infrared sensor for label identification, positioning and counting.

**Supplementary Information:**

The online version contains supplementary material available at 10.1007/s40820-022-00999-y.

## Graphical abstract

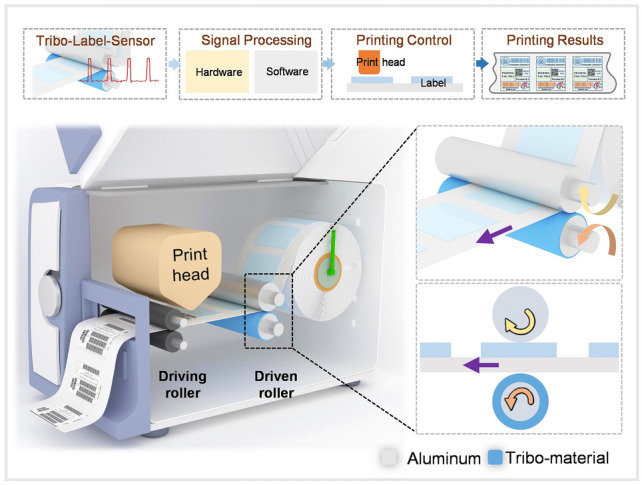

## Introduction

Label-printer has been extensively utilized in healthcare, biological medicine, logistics, retail, scientific research, radio frequency identification (RFID), etc*.* fields [1–3], and is becoming one of the sharpest swords for the development of Internet of Things (IoT) [4, 5]. During the printing process, label-sensor which used for label identification, positioning and counting provides feedback signal to the printer head and is the core component to ensure the proper function of the label printer. Commercially, various technologies have been developed for the label sensing, including infrared [6, 7], laser [8], capacitive [9] and ultrasonic [10] sensor. Among them, infrared label-sensor based on detecting the variation of reflected optical signal is the most commonly employed variant due to its high integrability, low cost and less power consumption [11]. However, there still exist some nonignorable drawbacks: (i) mechanical jitter of the label paper during fast printing would also be detected and disturb the useful signals; (ii) heat generated from long-time operation would affect the sensor performance; and (iii) transparent labels cannot be detected without pre-marking the black line on paper substrate. These disadvantages make the printer fail to realize high-speed recognition of labels as well as fast and stable printing.

Recently, originating from mechano-induced Maxwell displacement current [12–15], triboelectric nanogenerator (TENG) has been developed for converting mechanical movements into electricity with the superiorities of simple structure, flexibility, high integration, low cost and rich material selection [16–23]. In addition, owing to the saturated-output characteristic [24, 25], TENG as an active sensor holds ultra-high sensitivity to tiny displacement and has been demonstrated for detecting sphygmus [26], eye blinking [27], heart beating [28–32], breath [33, 34], touching [35, 36], microvibration [37], underwater acoustic [38], micro-fluidic [39], etc*.*, with the highest motion resolution in nanometer [40]. Harnessing these capabilities and the main structure of label printer, TENG technology could provide a simple and feasible solution for addressing the current critical issues facing in infrared label-sensor.

In this work, we propose a self-powered, highly sensitive and embedded tribo-label-sensor (TLS) for the fast and stable label printing. Based on the mechanism of contact-separation (C–S) mode TENG, TLS is designed on the intrinsic roller structure inside the label printer without changing the overall structure, which is a universal strategy for various label-printers. In the experiment, device parameters on sensing performance and deep comparison with infrared sensor are systematically investigated. As the results, TLS delivers 6 times higher signal magnitude than traditional one and is immune to label jitter and temperature variation during fast printing. Moreover, TLS can also be used for transparent label directly and keeps stable sensing performance after long-term operation. Additionally, a virtual-based software platform is successfully developed for demonstrating the potential capability of TLS in practical label printing. This reported TLS may have great potential to address some hardships in current label printer and further promote the development of IoT.

## Experimental

### Numerical Simulations

The potential distribution was numerically calculated using a commercial software COMSOL Multiphysics (5.6a version). The electrostatic field module was used to obtain all potential distributions at different separation distances under a 2D model.

### Fabrication of Tribo-Label-Sensor

The detailed fabrication process of TLS is shown in Fig. S1*.* Two aluminum rods of length 50 mm and diameter 6 mm were machined by lathe (WM210V, Yangzhou Acura Hardware Machinery Co., Ltd.) to form step shafts of length 5 mm and diameter 3 mm at the ends of each. Subsequently, one of the aluminum rods was covered with tribo-material. Finally, bearings were installed at the ends of each aluminum rod and connected to the external frame by preload of springs.

### Measurement and Characterization

A field-emission scanning electron microscope (Hitachi SU8010) was used to characterize the surface morphologies of FEP film. The electrical signals were recorded using a programmable electrometer (Keithley 6514). The operation speed of the label is 0.1 m s^−1^ driven by a linear motor (H01-23 × 166/180, LinMot). A fan was used to trigger random jitter of the label. Heat gun (GJ-8018LCD) was applied to heat the sensor surface and thermal imaging camera (IRay, T2S-A86) recorded the real-time temperature. The virtual label-printing interface was constructed based on LabVIEW, which could realize real-time data acquisition control, analysis and label printout display.

## Results and Discussion

### Structural Design and Working Principle

**Fig. 1:**
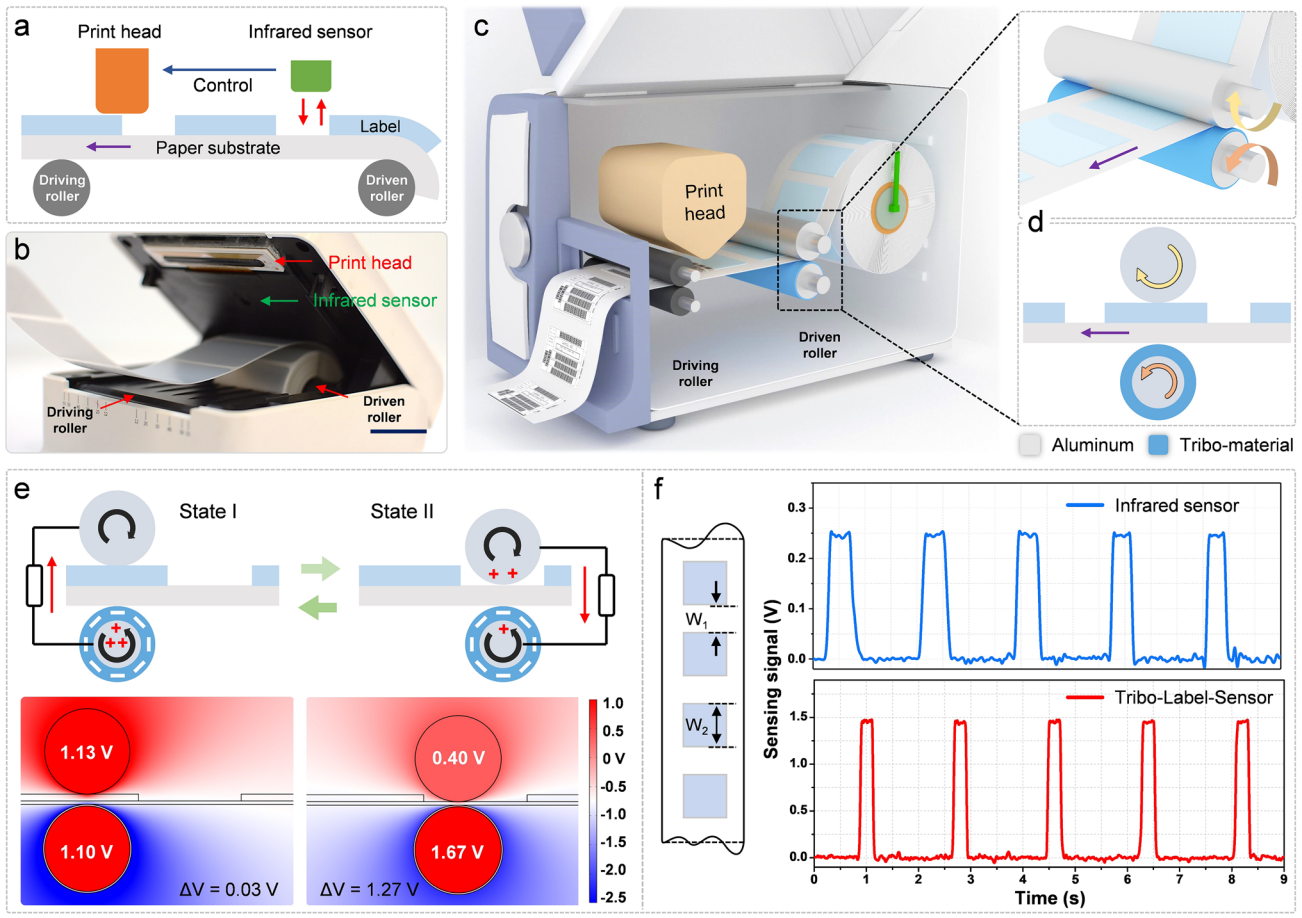
Structure and sensing mechanism of the tribo-label-sensor (TLS). **a** Schematic of the basic components during label printing. **b** Optical photograph of a commercial portable label printer (scale bar: 2 cm). **c** 3D schematic inside a label printer embedded with TLS. **d** The detailed structure and working status of TLS. **e** Charge (Up) and simulated potential (Down) distribution of TLS in two typical states under short-circuit and open-circuit condition, respectively. **f** Output sensing signal comparison of infrared sensor and TLS. *W*_1_ and *W*_2_ in left schematic represent the interval between two labels and the label width, respectively

For any label-printer, the basic label-printing processes and main components are schematically illustrated in Fig. 1a. Firstly, labels are evenly attached on the paper substrate and pushed forward by rotation of the driving roller. Subsequently, infrared label-sensor perceives the label position and provides feedback signal to control the print head operation. Figure 1b demonstrates the digital photograph of a commercially portable label-printer with the main components marked. The output sensing signal is generated due to the certain thickness of each label induced reflected infrared light variation when the interval of two labels passing through. Based on the sensing mechanism, label jittering caused by fast printing can be recorded and definitely affects the proper signal. Additionally, infrared sensor is hard to detect transparent label directly. Therefore, in this work, a tribo-label-sensor (TLS) is developed by embedding triboelectric nanogenerator (TENG) in the indispensable driving/driven roller of a label printer, as 3D schematic shown in Fig. 1c. The basic parts of TLS contain a lower conductive rod covered with tribo-material and upper conductive rod, and the label is pressed in between (Fig. 1d). The detailed fabrication of TLS used in the experiment is described in Fig. S1. With the labels moving forward, two rods cling to the label surface and sense the periodical gap variation caused by distributed labels. In this case, TLS could maintain stable sensing signal, be transparent label applicable, more efficient and integrated than infrared sensor. Figure 1e illustrates the working mechanism of electrical signal generation process for TLS, including the charge transfer schematic (top) and the potential distribution by finite element simulation (bottom). By rolling friction with paper substrate, fluorinated ethylene propylene (FEP) tribo-layer is negatively charged in the beginning stage (Fig. S2). The height difference between the label and paper substrate results in periodic approach and separation between the upper and the lower rod during printer operation. In state I, when the label moving through the roller, no charge distribution and potential difference vary as the distance between each TENG electrode keeps constant. In state II, when TLS is in the interval of labels, potential equilibrium of two electrodes is broken (open-circuit condition), and electrons flow from upper rod to lower rod (short-circuit condition), thus generating electric signal. Figure S3 shows the dynamic open-circuit voltage (*V*_OC_) and short-circuit current (*I*_SC_) output of TLS in one sensing period. As the printer operates continuously, state I and II revolve, generating periodical signals (Fig. 1f). Excitingly, for a given label with a certain label interval *W*_1_ (3 mm) and label width *W*_2_ (15 mm), the sensing signal of TLS is comparable to that of infrared sensor, even with higher magnitude.

### Basic Sensing Performance of TLS

**Fig. 2:**
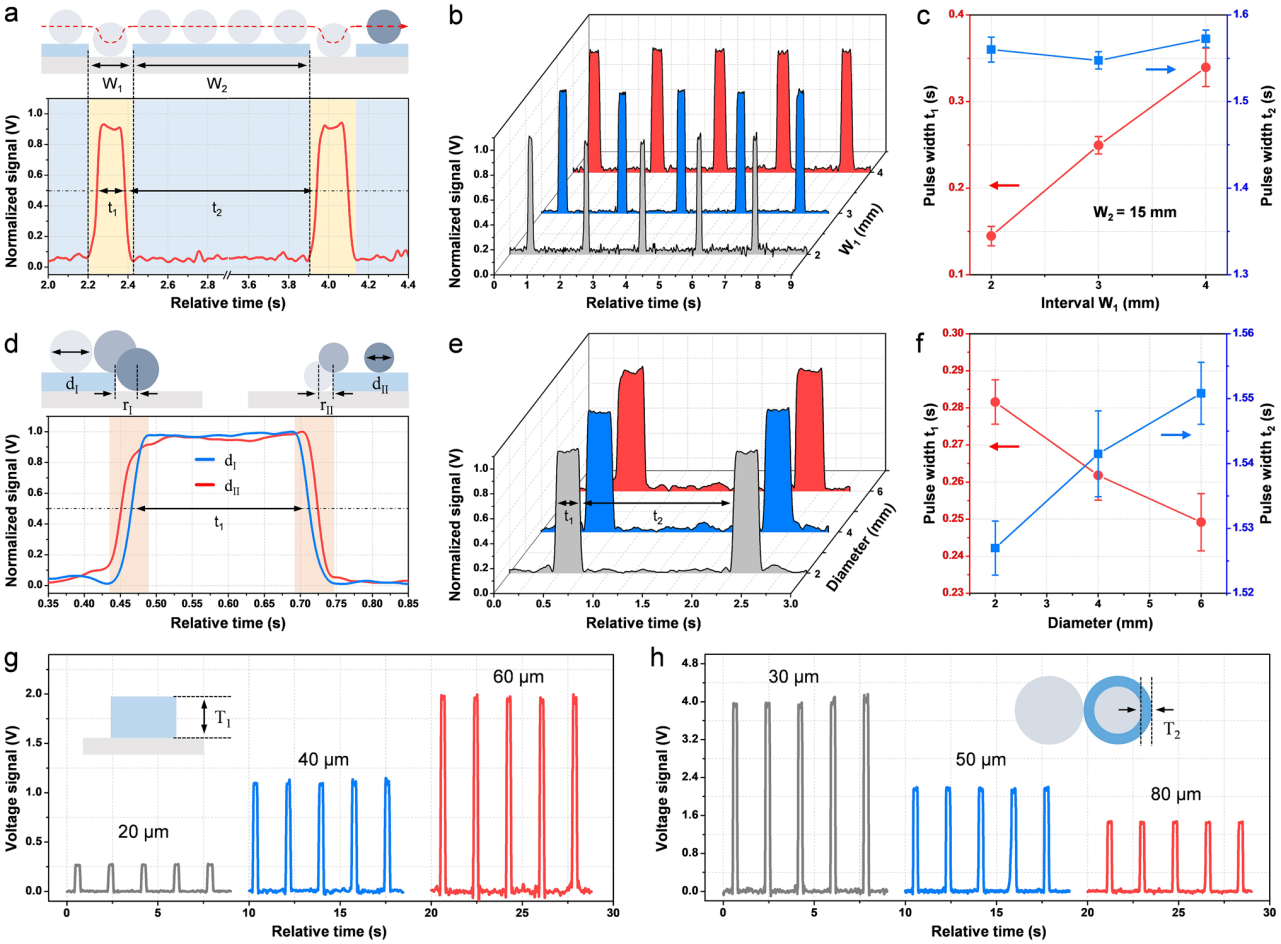
Basic performance characterization of TLS. **a** Schematic and dynamic output signal of a typical sensing process (*W*_1_ = 2 mm, *W*_2_ = 15 mm). **b** Sensing signals of TLS with different label intervals *W*_1_ and fixed label width *W*_2_ (15 mm). **c** Pulse time-width *t*_1_ and time-gap *t*_2_ of the sensing signal for different *W*_1_ (*W*_2_ = 15 mm). **d**, **e** Schematic and dynamic output signal of TLS with different roller diameters (*d*_I_ = 6 mm, *d*_II_ = 2 mm). **f** Pulse time-width *t*_1_ and time-gap *t*_2_ for different roller diameters. The influence of **g** label thickness and **h** tribo-layer thickness on the absolute output signal of TLS

In practice, the interval between two labels (*W*_1_) and the width of label (*W*_*2*_) are two basic label specifications, which need to be detected by the label-sensor. Figure 2a shows the relative motion trajectory of the rod and the corresponding normalized *V*_OC_ signal of TLS for a typical sensing process. It can be seen that the half-height time-width *t*_1_ of the pulse relates to the label-interval *W*_1_ and the label-width *W*_2_ is reflected by the time-gap *t*_*2*_ between adjacent pulse signals, respectively, which are determined by the sensing mechanism of TLS. Therefore, with label-interval *W*_1_ changing, the time-width *t*_1_ varies accordingly, as *V*_OC_ signal plotted in Fig. 2b and *I*_SC_ signal shown in Fig. S4. It is worth noting that the missing of labels can also be further analyzed from the time-width *t*_1_ (Fig. S5). Figure 2c quantitatively measures the correlation between *t*_1_, *t*_2_ and *W*_1_, *W*_2_, *t*_1_ shows excellent linearity to *W*_1_ (from 2 to 4 mm), and *t*_2_ keeps constant with the fixed *W*_2_ (15 mm). In addition, the output signal of infrared sensor for sensing the labels with different specifications is recorded in Fig. S6, which indicates that TLS holds the same sensing characteristic with the traditional one. Nevertheless, for the same *W*_1_, *t*_1_ measured by TLS is always smaller than that of infrared sensor (Fig. 1f). The reason is mainly based on the different sensing mechanism of each sensor (Fig. S7). In this case, for TLS, diameter of the roller would absolutely affect the sensing signal. It can be observed in Fig. 2d, e, the output *V*_OC_ curve of TLS with smaller rod diameter holds shorter rising or falling edges in pulse signal than that with larger diameter, since the smaller roller would cover more area of the interval during operation and achieve a higher sensing fidelity. As the quantitative result, with the rod diameter increasing, *t*_1_ decreases, while *t*_2_ increases linearly (Fig. 2f). For the label-sensor, sensitivity is also an important aspect, due to the mechanism of C–S TENG (Note S1), the output signal magnitude of TLS is highly related to the label thickness. As measured in Figs. 2g and S8, both *V*_OC_ and *I*_SC_ decrease with the label thickness decreasing. In order to improve the sensitivity of TLS, other than modification of tribo-material [41–44], the thinner tribo-layer is also contributive. As shown in Figs. 2h and S9, the sensing signal is stronger when using the thinner FEP layer. On one hand, based on the physical model of TENG [45–47], the thinner tribo-layer can effectively avoid electrostatic breakdown inner the device and further boost the maximum surface charge density (Fig. S9), which leads to the higher output magnitude. On the other hand, the output of C–S TENG will not keep increasing with the gap separation but has the saturated-output characteristic [24, 25]. In Fig. S10, the simulated results indicate that the saturated output can be reached more quickly with a thinner tribo-layer. The detailed discussion is presented in Note S1. Therefore, the thinner trio-layer brings higher surface charge density and more saturated output, which leads to the higher sensitivity. All above shows the capability of TLS for label sensing and comparability to infrared sensor.

### Performance Comparison with Infrared Sensor

**Fig. 3:**
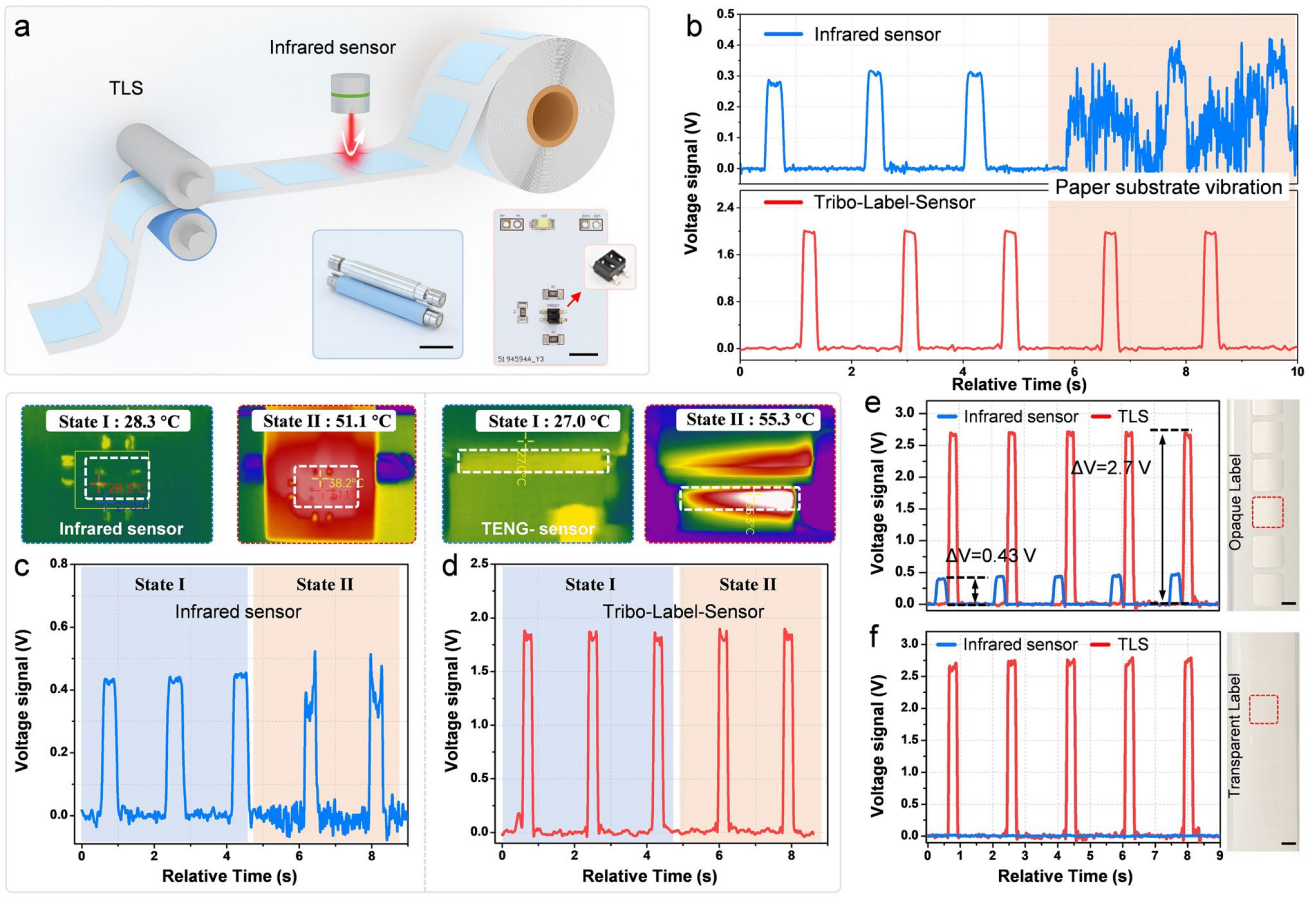
Performance comparison of TLS and infrared sensor. **a** The schematic of method and digital photographs of the sensors used for comparison (scale bar: 10 mm). The influence of **b** label jitter, **c**, **d** device temperature variation (upper parts are the thermal imaging of each sensor under difference working condition) and **e**, **f** label transparency on the output sensing signal of TLS and infrared sensor (scale bar: 10 mm)

Infrared sensor is most widely harnessed in label printer owing to its small size, high sensitivity and non-contact characteristic. To demonstrate the unique advantages of TLS, synchronous tests are carried out by comparing output signals of the front and rear-mounted sensors in one printing process, as schematically shown in Fig. 3a and experimental setup exhibited in Fig. S11. The insets of Fig. 3a show digital photographs of the TLS and infrared sensor used for the comparison, and the detailed circuit layout of the infrared one is illustrated in Fig. S12. From the integrability aspect, TLS is embedded in the roller, which needs no additional installation space and may further miniaturize the label printer. From the printing aspect, label jitter inevitably occurs during fast printing process, which triggers false localization of labels and consequently resulting in offset, skipped and off pages. Figure 3b shows the output signal comparison of TLS and infrared sensor under the influence of jitter. Obviously, the useful signal of infrared sensor is highly impacted and submerged in the interference signal by label jitter, while the sensing signal of TLS maintains excellent consistency, since the label is always pressed between two rollers, demonstrating its immunity to mechanical interference. In addition, long-term printer operation generates heat and causes temperature variation, which has influence on the performance of most electronic elements. The infrared thermal imaging photos and the signals of infrared sensor and TLS before and after heating under the same sensing process are exhibited in Fig. 3c, d, respectively. The output signal of TLS keeps stable at both state I and state II, the signal of infrared sensor is stable at state I but becomes fluctuant at state II, indicating the thermal stability of TLS is higher than traditional one. Furthermore, due to the basic sensing principle, the performance of infrared sensor highly depends on label materials. As shown in Fig. 3e, TLS and infrared sensor both generate valid signals for the silvery opaque label. Here, the signal magnitude of TLS with 50 μm tribo-layer is nearly 6 times than infrared one, and it can be further increased by a thinner tribo-layer. This high sensitivity may greatly simplify the signal processing circuit of the printer. For transparent label which is also commonly used in RFID area, there is no output signal sensed by infrared sensor, while TLS still keeps the valid output signal (Fig. 3f). All the above proves that TLS has superior advantages than infrared sensor in integrability, sensitivity, antijamming and universality for label printer. In addition, Fig. S13 shows the optical photographs of the label surface before and after an operation of the TLS. It is worth noting that no scratches are observed.

### Application Feasibility of TLS

**Fig. 4:**
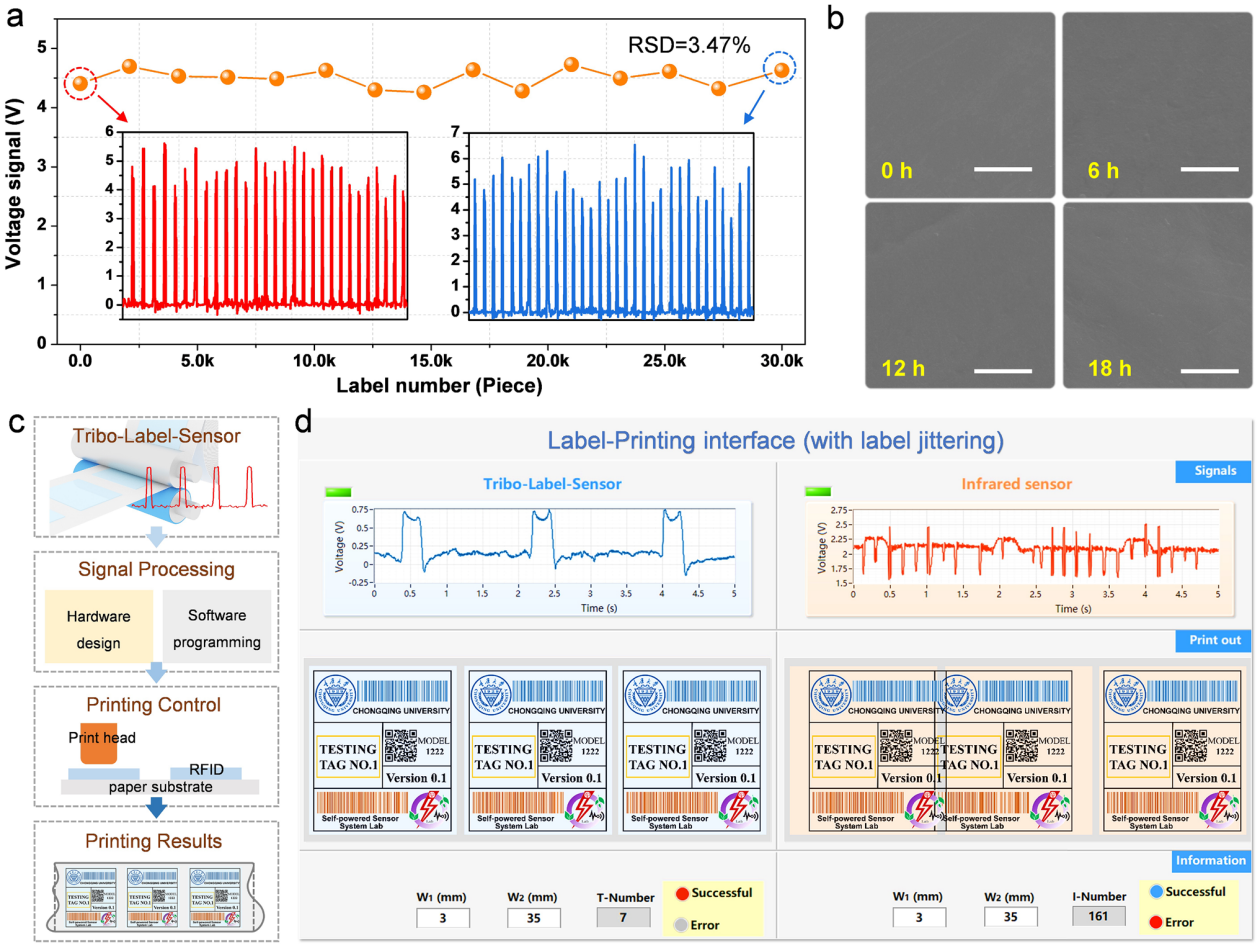
Demonstration of application feasibility of TLS. **a** Long-term stability of TLS after 30,000 cycles. The insets show the detailed sensing signal at the initial and final stage. **b** Surface morphology of the FEP film after operating for 0, 6, 12, and 18 h. **c** Schematic diagram of the workflow of a label printer based on TLS. **d** Label printing demonstration using TLS and infrared sensor based on the LabVIEW virtual platform

For the label-sensor, robustness is also the significant aspect for realizing long-time stable and reliable label printing, but is the critical facing issue of TENG that needs to be addressed. Fortunately, the static rolling friction and contact-separation mode in the roller-structured TLS would largely avoid mechanical abrasion and ensure high durability. As data recorded in Fig. 4a, the signal magnitude of TLS almost maintains constant (slightly fluctuates) during 30,000 cycles’ long-term operation. Figure 4b shows the SEM images of the FEP surface; no significant scratches are observed after 6, 12 and 18 h of continuous testing, which further proves the feasibility of TLS in practical application. To build a label printing system, the workflow using TLS is similar to that of infrared sensor, with a pre-set phase difference between the sensor and the print head, the real-time sensing signal controls the print head after signal processing to achieve accurate printing position, as schematically illustrated in Fig. 4c. Finally, a virtual label-printing interface was developed to demonstrate the system feasibility of TLS for properly operating under the known interference (Fig. S14). Without disturbance, TLS and infrared sensor both successfully implement label printing (Video S1). While, with disturbance, the output signal of infrared sensor is swamped by the interference, resulting in misaligning and overlapping of printed labels (Fig. 4d). On the contrary, TLS remains stable signal and correct label printing results (Video S2). All above proves the application feasibility of TLS as an alternative toolkit with outstanding advantages for practical label printing.

## Conclusion

In summary, based on embedding triboelectric nanogenerator into the intrinsic roller structure of the label printer, we have developed a self-powered and highly sensitive tribo-label-sensor for the fast and stable label printing. The sensing mechanism and device performance were systematically investigated both in theory and experiment. Comparing to traditional infrared sensor, TLS possessed even higher integrability for printer miniaturization, much higher signal magnitude (over 6 times), and can detect transparent label directly. The fidelity and sensitivity of TLS could be further improved by roller parameter and tribo-layer optimization. Moreover, the mechanical jitter and generated heat during continuously fast printing showed no interference to the sensing signal of TLS. To demonstrate the application feasibility, TLS maintained both excellent electric and mechanical durability after 30,000 operation cycles. The potential capability of TLS in practical label printing was also successfully realized using a virtual-based software platform. The superior advantages to infrared sensor in self-powered, integrability, sensitivity, antijamming and universality clearly present TLS as an outstanding alternative toolkit to further promoting label printer technology and IoT.

### Supplementary Information

Below is the link to the electronic supplementary material.

Supplementary file1 (MP4 22456 KB)

Supplementary file2 (MP4 15329 KB)

Supplementary file3 (PDF 1241 KB)
