# Supplementary material for: A Self-Powered, Highly Embedded and Sensitive Tribo-Label-Sensor for the Fast and Stable Label Printer
Source: Nanomicro Lett. 2022 Dec 31;15:27. doi: 10.1007/s40820-022-00999-y (PMC9805486; doi:10.1007/s40820-022-00999-y)
Supplement: Supplementary file 3 — Supplementary file3 (PDF 1241 KB) [file 40820_2022_999_MOESM3_ESM.pdf]

Supporting Information for

A Self-Powered, Highly Embedded and Sensitive Tribo-Label-Sensor for the Fast and Stable Label Printer

Xindan Hui[1], Zhongjie Li[2], Lirong Tang[1], Jianfeng Sun[1], Xingzhe Hou[5], Jie Chen[4], Yan Peng[2, *], Zhiyi Wu[3, *] and Hengyu Guo[1, *]

[1] School of Physics, Chongqing University, Chongqing, P. R. China

[2] School of Mechatronic Engineering and Automation, Shanghai University, Shanghai, P. R. China

[3] Beijing Institute of Nanoenergy and Nanosystems, Chinese Academy of Sciences, Beijing, P. R. China

[4] College of Physics and Electronic Engineering, Chongqing Normal University, Chongqing, P. R. China

[5] Electric Power Research Institute, State Grid Chongqing Electric Power Company, Chongqing, P. R. China

* Corresponding authors. E-mail: physghy@cqu.edu.cn (H. Guo); pengyan@shu.edu.cn (Y. Peng); wuzhiyi@binn.cas.cn (Z. Wu)

Supplementary Note S1

The basic contact-separation model of TENG is shown in Fig. S10a. The thickness of the dielectric material is d_1, the charge density is $-\sigma$, and the distance between the two triboelectric materials during contact separation is *x* as a function of time. The total charge on electrode II is ($S\sigma$-Q), the former is the total charge generated by triboelectric effect, and the latter is the charge transferred between the two electrodes in the process of electrostatic induction. Ignoring the boundary effect, the potential difference between the two electrodes can be expressed as:

$$V = E_1 d_1 + E_{air} x = -\frac{Q}{S\varepsilon_0}\left(\frac{d_1}{\varepsilon_{r1}} + x(t)\right) + \frac{\sigma x(t)}{\varepsilon_0} \quad \text{(S1)}$$

where ε_0 is the vacuum permittivity and ε_{r1} is the relative permittivity of tribo-material. Define the effective thickness:

$$d_0 = \sum_{i=1}^{n} \frac{d_i}{\varepsilon_{ri}} \quad \text{(S2)}$$

Combining Eq. (S2) and Eq. (S1), then

$$V = -\frac{Q}{S\varepsilon_0}\left(d_0 + x(t)\right) + \frac{\sigma x(t)}{\varepsilon_0} \quad \text{(S3)}$$

Deriving from Eq. (S3):

$$V_{OC} = \frac{\sigma S}{\varepsilon_0} \quad \text{(S4)}$$

$$Q_{SC} = \frac{S\sigma x(t)}{d_0 + x(t)} \quad \text{(S5)}$$

From Eq. (S5), it can be seen that, the charge Q_{SC} is related not only to the separation distance between the two surfaces, but also to the thickness of the tribo-layer. With a fixed separation distance and surface charge density, the transferred charge Q_{SC} would increase as the thickness of tribo-layer decrease.

The relationship between the charge Q_{SC} and the separation distance for different thicknesses of the tribo-layer is demonstrated by using Comsol Multiphysics software simulation, as shown in Fig. S10b. As the thickness of tribo-layer is kept at a constant value, the output charge Q_{SC} gradually increases with the separation distance and eventually tends to saturate because the charge transfer between the two electrodes is almost non-existent after the separation distance increases beyond a certain value. As the thickness of the tribo-layer changes, the charge transfer of the thinner tribo-layer tends to saturate more rapidly, in other words, at the same separation distance, more output is obtained with the thinner tribo-layer, which is consistent with the derivation of Eq. (S5).

Supplementary Figures

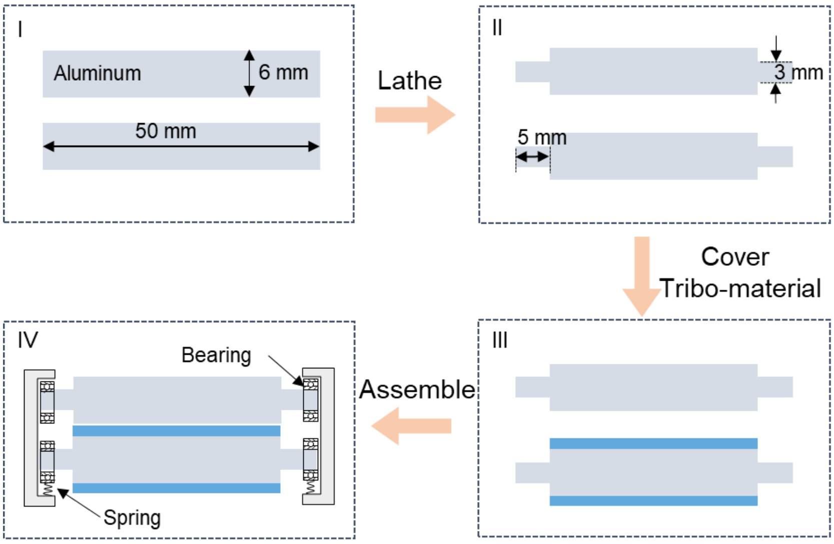

Fig. S1 Fabrication process of TLS

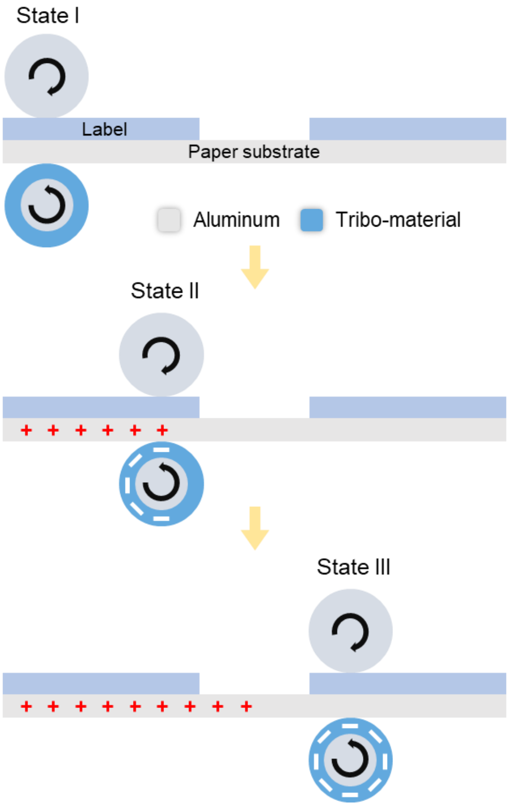

Fig. S2 Triboelectrification process at the beginning stage

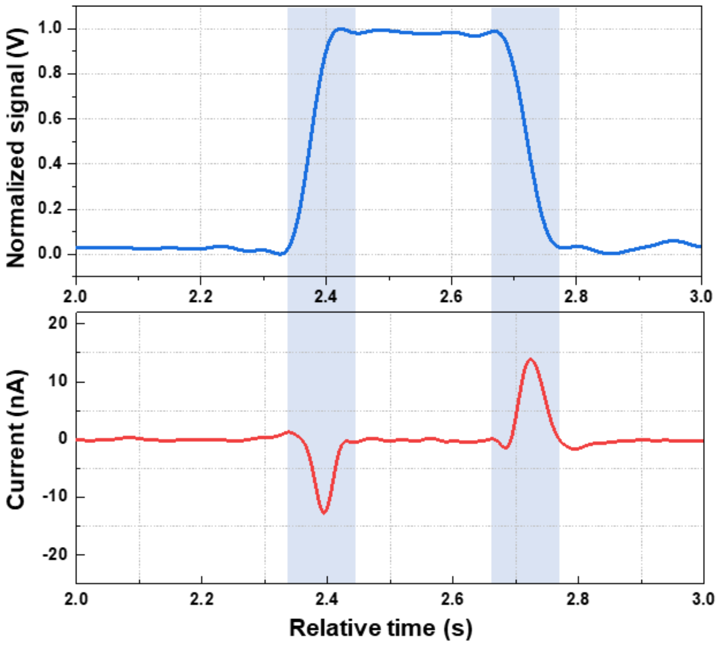

Fig. S3 Dynamic open-circuit voltage and short-circuit current output of TLS in one sensing period

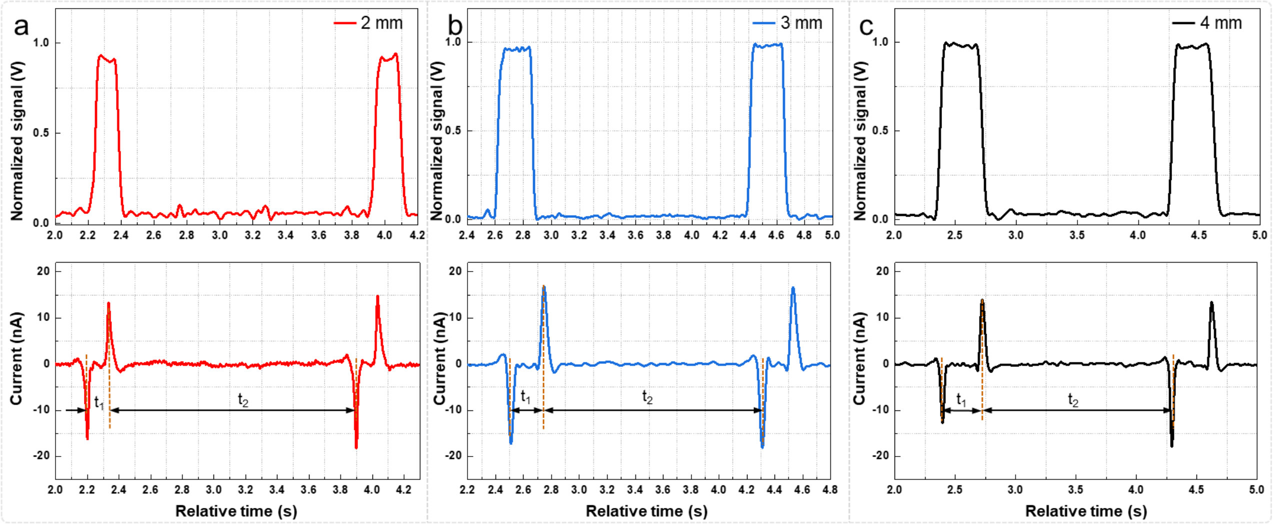

Fig. S4 Output voltage and corresponding current signal of TLS for label sensing with different label-interval W_1 (W_2 = 15 mm)

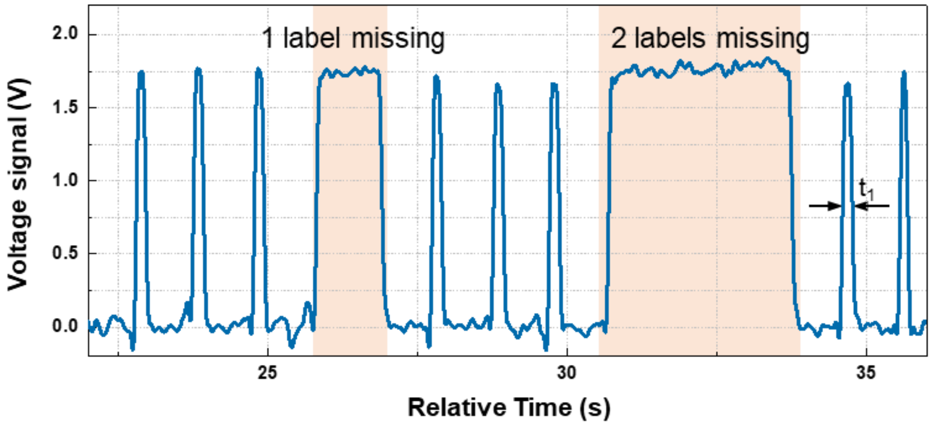

Fig. S5 Sensing signal of TLS for labels with missing pieces

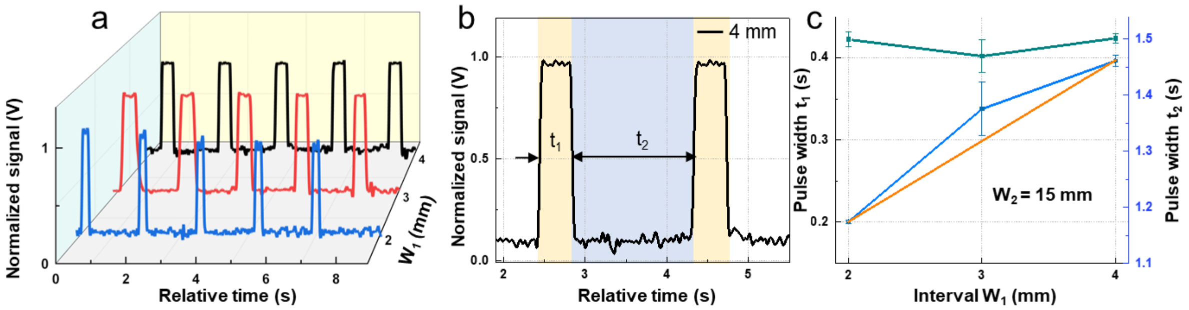

Fig. S6 Signal characteristics of infrared sensor for sensing the labels with different specifications

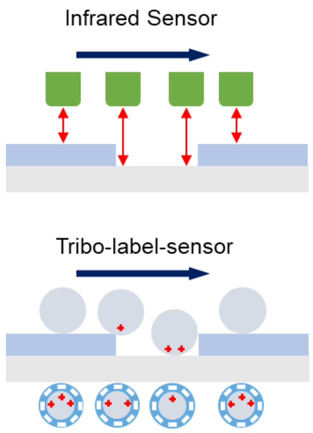

Fig. S7 Dynamic sensing process of infrared sensor and TLS

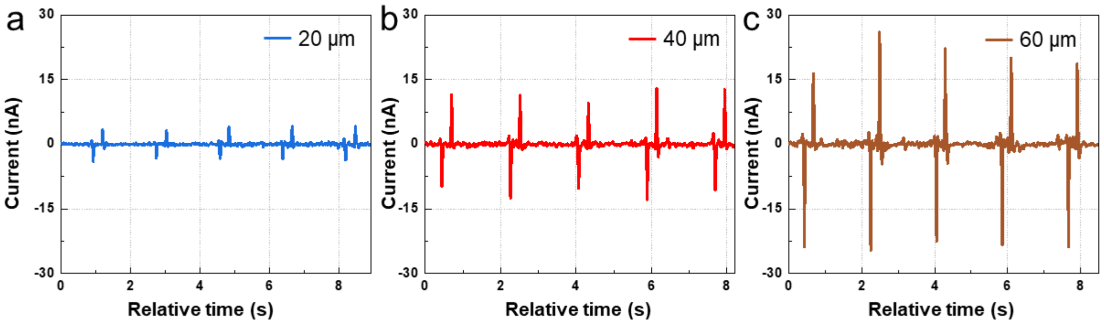

Fig. S8 Short-circuit current of TLS with the label thickness varying

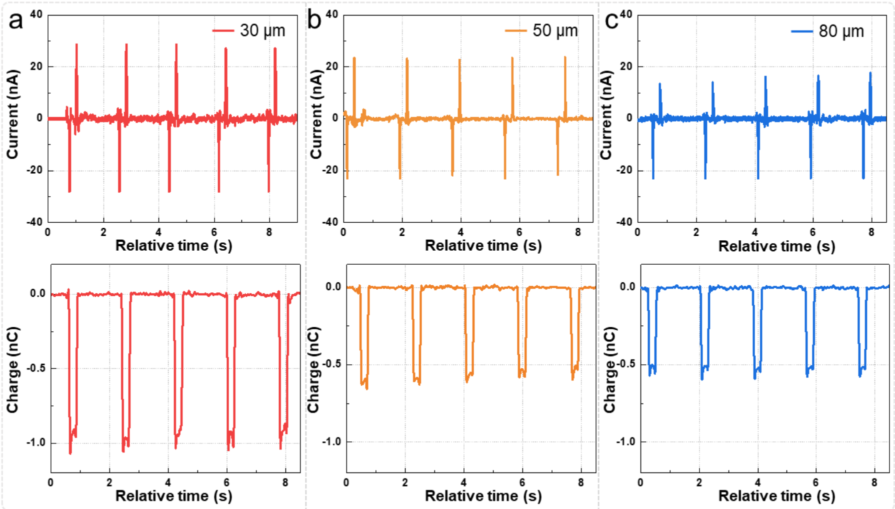

Fig. S9 Current and charge output of TLS with various thickness of tribo-layer

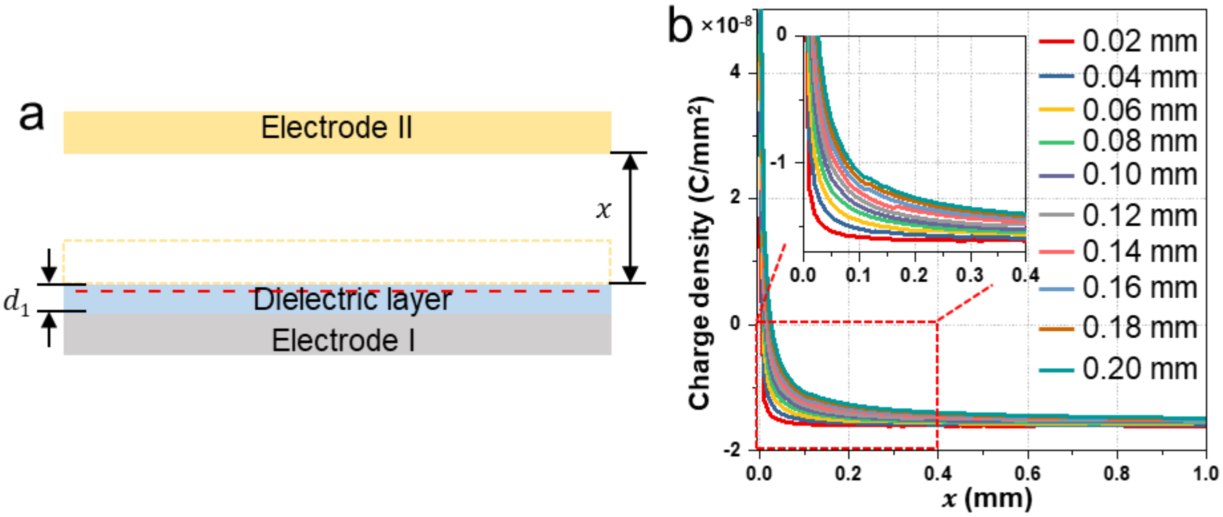

Fig. S10 The simulated charge transfer of C-S TENG with different thickness of tribo-layer under the same surface charge density

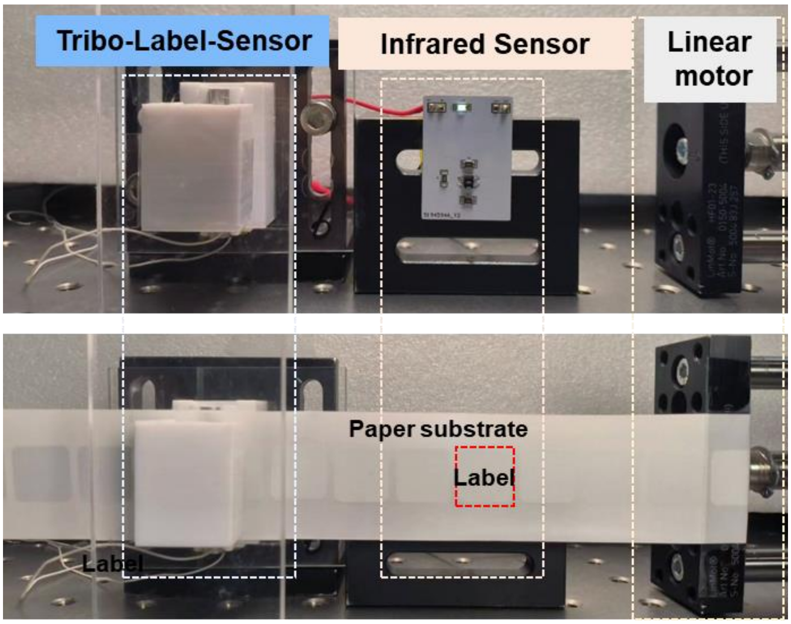

Fig. S11 Digital photograph of the experimental setup for device performance comparison

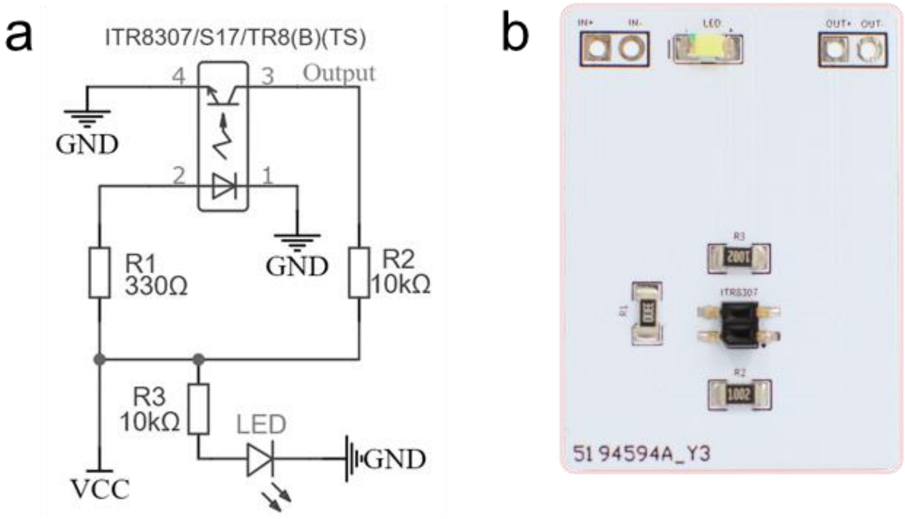

Fig. S12 Detailed circuit layout and image of the infrared sensor for testing

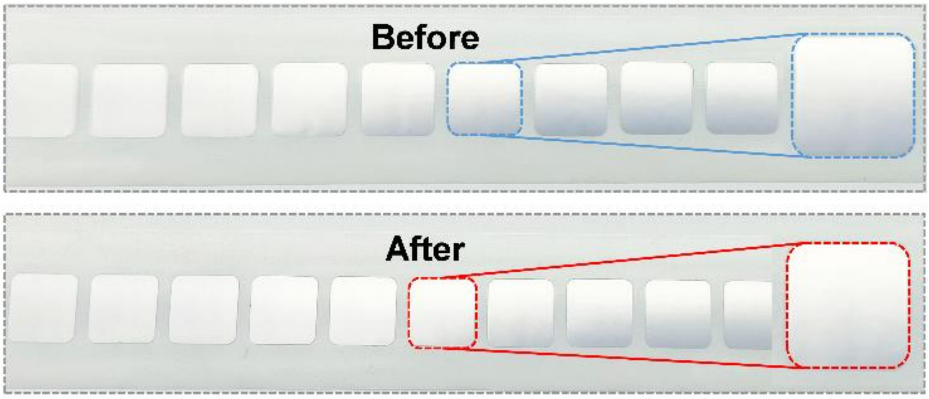

Fig. S13 The optical photographs of the label surface before and after an operation of TLS

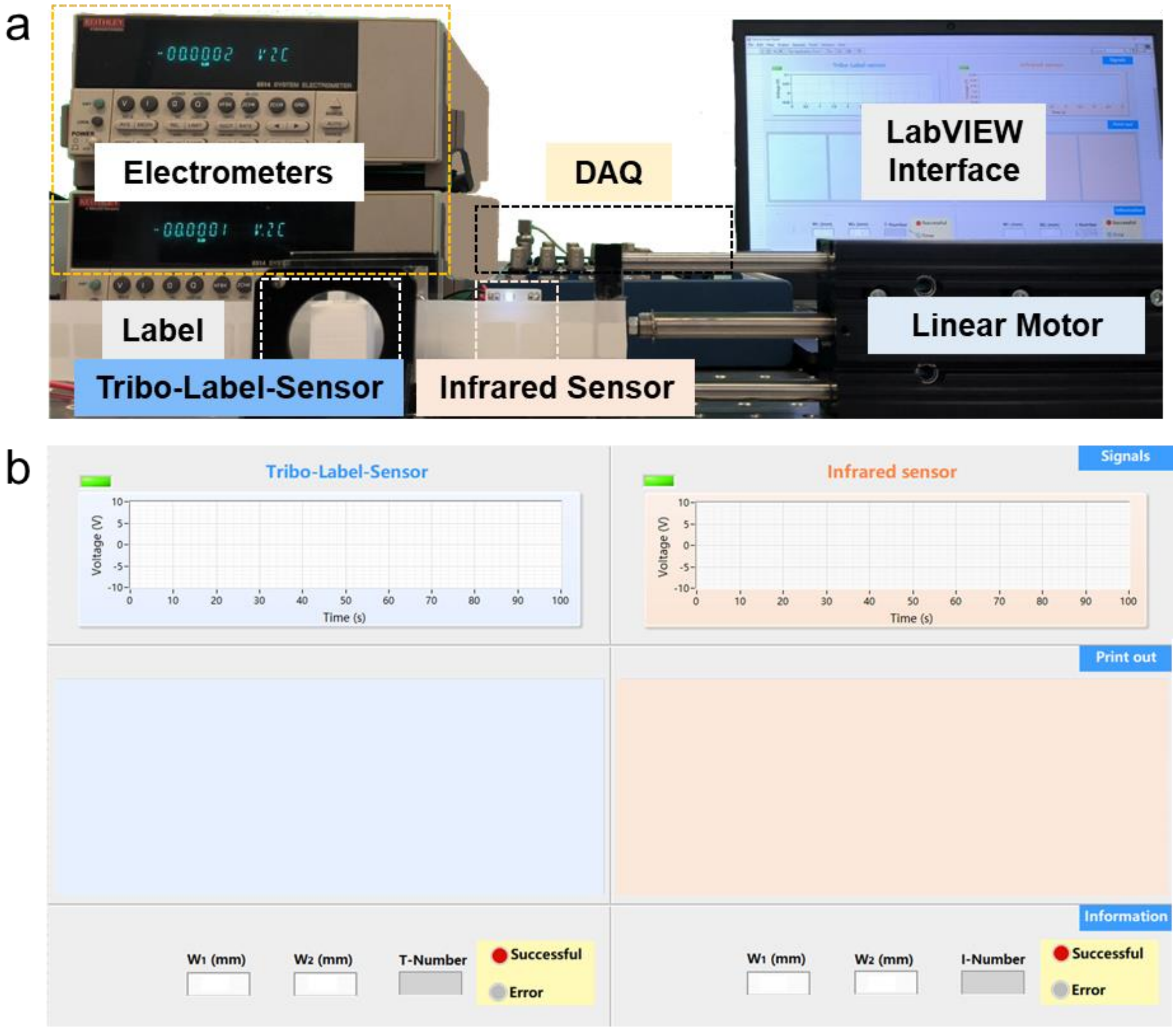

Fig. S14 A virtual label printing interface based on LabVIEW platform
